# Reduced workload demand with handheld robotic‐assisted revision knee arthroplasty: A multi‐centre NASA Task Load Index analysis

**DOI:** 10.1002/jeo2.70737

**Published:** 2026-04-30

**Authors:** Andrew Porteous, James Murray, Max Ettinger, Peter Savov, Matteo Innocenti, Gijs van Hellemondt, Koen DeFoort, Juan Carlos Martinez‐Pastor, Peter Bollars, Francesco Zambianchi, Alberto Belluati, Catherine Whittall

**Affiliations:** ^1^ Southmead Hospital Bristol UK; ^2^ Pius Hospital Oldenburg Germany; ^3^ Azienda Ospedaliero‐Universitaria Careggi, Università degli Studi di Firenze Florence Italy; ^4^ Sint Marteenskliniek Nijmegen The Netherlands; ^5^ Hospital Clinic Barcelona Spain; ^6^ Sint‐Trudo Ziekenhuis Sint‐Truiden Belgium; ^7^ Azienda Ospedaliero‐Universitaria di Modena Università degli Studi di Modena e Reggio‐Emilia Modena Italia; ^8^ Ospedale S. Maria delle Croci Ravenna Italy; ^9^ Smith and Nephew Watford UK

**Keywords:** cognitive demand, NASA‐TLX, revision knee surgery, robotic‐assisted knee arthroplasty, surgical workload reduction

## Abstract

**Purpose:**

Revision total knee arthroplasty is a complex and technically demanding procedure, with an increasing global incidence. This complexity heightens the cognitive and physical demands of surgical teams, contributing to elevated burnout, musculoskeletal injuries and psychological stress. This European wide multi‐centre observational evaluation assessed the impact of a handheld robotic‐assisted surgical system on intra‐operative workload using the NASA Task Load Index instrument to compare robotic‐assisted and manual instrumentation approaches during revision knee arthroplasty.

**Methods:**

Data were collected from 212 surveys following 100 revision knee arthroplasty procedures, capturing subjective workload measures across six NASA Task Load Index domains from surgeons and peri‐operative theatre staff.

**Results:**

The results demonstrated a significant reduction in the overall workload with robotic‐assistance compared with manual instrumentation (median overall workload score 0.0 vs. 8.5 respectively, *p* = 0.0003). Domain‐specific analyses revealed significantly lower mental demand, physical demand and effort in the robotic‐assisted group without compromising perceived surgical performance, temporal demand, or frustration. A subgroup analysis of the lead surgeons showed consistent perceived workload reductions consistent with the overall findings. These results suggest that the handheld robotic‐assisted surgical system may mitigate cognitive and physical strain during complex revision knee arthroplasty procedures.

**Conclusions:**

This evaluation underscores the potential of robotic‐assisted technology to enhance surgical ergonomics, reduce surgeon fatigue and improve well‐being, which may translate into sustained surgical performance. Future work should incorporate objective ergonomic measures, expand participant roles and explore long‐term impacts on clinical outcomes and staff welfare.

**Level of Evidence:**

Level II.

**Abbreviations**
IQR: interquartile range
NASA‐TLX: NASA Task Load Index
OWS: overall workload score
RA: robotic‐assisted
RKCC: revision knee complexity classification
rTKA: revision total knee arthroplasty
SD: standard deviation
TKA: primary total knee arthroplasty
UK: United Kingdom
US: United States

## INTRODUCTION

Revision total knee arthroplasty (rTKA) is one of the fastest growing and most technically demanding segments of orthopaedic surgical practice, with international registries projecting a substantial increase in revision procedures as the population ages and earlier joint replacement becomes more common. Estimates from the United States forecast an increase in rTKA of 149% by 2040 and 520% by 2060 [29]. Similarly, in the United Kingdom, rates are projected to rise by 332% by 2030 [23]. Unlike primary TKA, revision surgery is complicated by factors such as bone loss, altered anatomy, multiple prior incisions, and the need for component removal/extraction, all of which increase the surgical complexity and extend the overall operation time. This accumulated stress has meaningful downstream consequences: by the time the reconstruction phase begins, when precision, focus and strategic decision‐making are crucial, the team may already be operating with diminished performance capacity. Consequently, the complexity of rTKA does not only influence patient outcomes, but also directly affects the wellbeing, endurance and cognitive efficiency of the surgical team, especially when these demanding procedures are performed repeatedly within congested operating schedules [14].

Emerging data across global orthopaedic communities highlight that burnout, musculoskeletal injuries and psychological stress are disproportionately high among surgeons engaged in complex arthroplasty, especially trauma and revision cases [5, 6, 21, 27, 30, 31, 32, 33]. Systematic reviews consistently demonstrate high burnout rates worldwide, for example exceeding 30% in French orthopaedic surgeons, 65% in Spanish surgeons and upwards of 90% among UK consultants [6, 31]. Forty per cent of French orthopaedic surgeons deemed their stress levels to be unacceptable [31]. There is an established association between high case complexity, physical fatigue and the risk for chronic pain and occupational injury, not only among surgeons but also across nursing and perioperative staff who directly support these procedures [18].

Traditionally, rTKA relies on manual instrumentation and the surgeons' judgement for balancing gap restoration, bone resection and implant alignment, factors that are frequently compromised by previous surgical interventions and patient‐specific anatomical challenges. Robotic‐assisted (RA) surgical systems, initially validated in primary TKA, can offer advanced intraoperative 3D mapping, real‐time balancing and quantitative performance feedback. The handheld CORI™ surgical system (Smith & Nephew, Inc.) assists surgeons with rTKA [11] without the need for pre‐operative imaging, which saves time, reduces costs and removes barriers posed by pre‐existing implants or distorted anatomy [7]. These benefits have played a key role in its adoption into routine clinical practice, often replacing conventional techniques.

Assessment of the cognitive, physical and emotional workload in surgical teams has become a priority for improving staff welfare and patient safety. The NASA Task Load Index (NASA‐TLX) provides a validated cross‐disciplinary framework for quantifying these demands, capturing nuanced differences across domains such as perceived performance success, mental demand, physical demand, temporal demand, effort and frustration [3, 8, 13, 17]. To date, although the literature suggests a 176% increase in the workload demand on surgeons for rTKA, compared to TKA [22], there remains a critical gap in that there is no published literature that examines the real‐world impact of RA versus conventional instrumentation on the workload of surgeons or the wider surgical teams in the setting of rTKA. The current evaluation addresses this gap using a multi‐centre survey and the NASA‐TLX instrument, directly comparing RA and manual surgical approaches.

This evalution aimed to evaluate the impact of a handheld RA surgical system on cognitive, physical and mental workload, perceived surgical performance, effort and frustration levels among operating theatre staff during rTKA.

## METHODS

A European wide multi‐centre observational evaluation was conducted with orthopaedic surgeons and operating theatre teams completing rTKA procedures with either manual instrumentation or RA. The use of the handheld RA surgical system was not based on clinical guidelines or policies but rather, dictated by the lead surgeon's preference and experience with the technology.

Participation in the survey was entirely voluntary, with lead surgeons, trainee surgeons, scrub nurses and circulating nurses invited to complete it after every rTKA surgery. No identifiable personal information was collected, other than the participants' professional role in the procedure. Responses were anonymised to ensure confidentiality. In accordance with institutional guidelines and local regulations, ethical approval was not required for this observational survey.

The survey was provided to participants by way of a QR code or a web‐link, and was hosted on the SNAP software platform (Snap Surveys) which incorporated the NASA‐TLX instrument [13] to capture self‐reported workload scores post‐procedure. The NASA‐TLX portion of the survey consisted of 6 domain‐specific questions on a 20‐point scale (see Table 1). Alongside the NASA‐TLX questions, the participants' experience with the handheld RA surgical system was captured to determine how far along they were with their learning curve, with a range from 1 to 4, 5 to 9, 10 to 14, 15 to 20 and >20 cases completed with the system. We anticipated that the steepest changes in workload would occur during the first 20 cases. We therefore divided this range into narrower bands (1–4, 5–9, 10–14, 15–19) to capture potential non‑linear improvements in workload demand. The ≥20 category was chosen to represent surgeons with more consolidated experience. For cases completed with manual instrumentation, the surgeon's experience with the handheld RA surgical system was still captured, the number of procedures they had previously completed with manual instrumentation was not included. The complexity of the rTKA procedure was determined by the lead surgeon based on the revision knee complexity classification (RKCC) [24], in which, cases were reported as R1 (least complex), R2 (complex), or R3 (most complex or salvage cases). The participants indicated their role and selected the time of day the procedure was started (morning, before 12 p.m.; afternoon, 12 p.m.–5 p.m.; evening, after 5 p.m.). A free text field was available so participants could report if there were any complications that arose during the procedure.

**Table 1:** NASA Task Load Index survey questions and responses.

Domain	Question	Response
PERFORMANCE	How successful were you in accomplishing what you were asked to do?	+10: Very high, perfect −10: Very low, failure
MENTAL DEMAND	How mentally demanding was the task?	+10: Very high −10: Very low
PHYSICAL DEMAND	How physically demanding was the task?	+10: Very high −10: Very low
TEMPORAL DEMAND	How hurried or rushed was the pace of the procedure?	+10: Very high −10: Very low
EFFORT	How hard did you have to work to accomplish your level of performance	+10: Very high −10: Very low
FRUSTRATION	How insecure, discouraged, irritated, stressed and/or annoyed were you?	+10: Very high −10: Very low

### Statistical analysis

In this analysis, the mean NASA‐TLX domain responses and overall workload scores (OWS) are evaluated. The OWS is comprised of a weighted sum of the six NASA‐TLX domains, calculated by reversing the performance score, adding it to the remaining five domain scores, and dividing by six (scaled from −45 [worst] to +50 [best]). A subgroup analysis of the data from the lead surgeons was completed for the mean NASA‐TLX domain scores and the OWSs.

GraphPad Prism (version 10.5.0 for Windows, GraphPad Software LLC) served as the platform for all statistical analyses. Data normality was assessed using the Shapiro–Wilk test. Group comparisons for nonparametric data were examined using Mann–Whitney *U* tests presented as medians with inter‐quartile ranges (IQR). Parametric data were assessed with a Student's *t*‐test and are presented as means ± standard deviation (SD). Multiple group analyses of nonparametric data were completed using the Kruskal–Wallis test with Dunn's multiple comparisons post‐test and presented as medians with IQR. Statistical significance was defined as *p* < 0.05.

### Statistical justification for sample size

A post‐hoc power analysis was performed with an alpha level of 0.05 and a target statistical power of 0.80. Using observed group means and standard deviations within an unpaired *t*‐test framework, the analysis confirmed that the achieved sample size satisfied the minimum requirement for detecting clinically and statistically meaningful differences in the primary outcome measures. A total of 136 survey responses (68 per group) provided sufficient power to detect differences in workload scores.

## RESULTS

### Data collection

Overall, 212 surveys were completed following 100 rTKA procedures (39 from manual instrumentation, 61 RA cases) for this analysis, a breakdown of survey responses by role is described in Table 2.

**Table 2:** Survey responses based on procedure variables.

Role	Manual instrumentation (*n*, %)	Robotic assistance (*n*, %)
Lead surgeon	39 (41.5)	61 (51.7)
Trainee surgeon	27 (28.7)	25 (21.2)
Scrub nurse	18 (19.1)	21 (17.8)
Circulating nurse	10 (10.6)	10 (8.5)
Revision knee complexity classification		
R1: Least complex	6 (6.4)	30 (25.4)
R2: Complex	59 (67.8)	72 (61.0)
R3: Most complex or salvage	29 (30.9)	14 (11.9)
Learning curve		
1–4	38 (40.4)	43 (36.4)
5–9	20 (21.3)	31 (26.3)
10–14	8 (8.5)	13 (11.0)
15–20	7 (7.4)	14 (11.9)
>20	25 (26.6)	22 (16.8)
Total survey response	94	118
Surgery start time^a^		
Morning (before 12 p.m.)	60 (70.6)	42 (57.5)
Afternoon (12 p.m.–5 p.m.)	22 (25.9)	27 (37.0)
Evening (after 5 p.m.)	6 (7.1)	7 (9.6)
Total surgery start time responses	85	73

^a^ Surgery start time was added to the survey post‐launch therefore only completed for 158 of the surveys.

The procedure variables are described in Table 2. The proportion of least complex responses was higher in the RA group (25.4% vs. 6.4%) and the percentage of responses for the most complex cases was 30.9% for manual instrumentation compared to 11.9% with RA.

Within the staff involved, there are varying ranges of experience with the handheld RA surgical system, some are earlier in their learning curve than other users of the system. For both groups, most survey responses were from staff who were in the earliest phase of their learning curve with the handheld RA surgical system (1–4 cases, 40.4% of respondents in the manual group and 36.4% in the RA group). In both cohorts, the largest proportion of surgeries were completed in the morning, before 12 p.m. (70.6% and 57.5% for manual and RA respectively, compared to afternoon surgeries (manual instrumentation 25.9%; RA 37.0%). There were a smaller number of cases in both groups that were conducted in an evening, after 5 pm (7.1% manual; 9.6% RA).

### NASA‐TLX domains

Domain‐specific analysis revealed that rTKA with the handheld RA surgical system was associated with significantly lower mental demand, physical demand and effort (*p* < 0.0001, Table 3). There were no significant differences between groups for performance, temporal demand or frustration.

**Table 3:** Kruskal–Wallis with Dunn's post‐test analysis of NASA‐TLX domains (*p* = <0.0001) and subgroup analysis of lead surgeons (*p* ≤ 0.0001).

	Manual instrumentation median (IQR)	Robotic assistance median (IQR)	*p*
Performance	7 (5–8)	8 (6–9)	>0.9999
Mental demand	5 (3–7)	3 (0.75–5)	**0.0065**
Physical demand	4.5 (2.75–6)	1.5 (0–4)	**<0.0001**
Temporal demand	2 (0–5)	2 (−2 to 5)	>0.9999
Effort	5 (3–7)	4 (1.5–6)	**0.0214**
Frustration	0 (−5.25 to 3)	0 (−5 to 2)	>0.9999
Subgroup analysis: Lead Surgeons			
Performance	7 (6–8)	8 (7–0)	0.4781
Mental demand	5 (3–7)	2 (0–5)	**0.0020**
Physical demand	6 (4–6)	0 [−2.5 to 3.5)	**<0.0001**
Temporal demand	3 (0–5)	1 (−3 to 4.75)	>0.9999
Effort	5 (4–8)	3 (1–6)	**0.0028**
Frustration	2 (−4 to 5)	−2 (−6.5 to 1)	0.0503

*Note*: Statistically significant differences are highlighted in bold (*p* < 0.05).

Abbreviation: IQR, interquartile range.

Among lead surgeons, domain‐specific analysis revealed a similar trend, with significantly lower mental demand, physical demand and effort observed when procedures were performed using RA (*p *≤ 0.0001, Table 3). No significant differences were detected in perceived performance, temporal demand or frustration levels.

### Overall workload scores

Compared to rTKA performed with manual instrumentation, the use of the handheld RA surgical system led to an 8.5‐point reduction in the median OWS (8.5 (IQR −4.0 to 15.0) versus 0.0 (IQR −9.0 to 9.8), *p* = 0.0003). A subgroup analysis of the data from the lead surgeons revealed a significant reduction of 13.5‐points in the mean OWS, decreasing from 9.4 ± 11.9 with manual instrumentation to −4.1 ± 14.3 (*p* ≤ 0.0001) with RA.

## DISCUSSION

To our knowledge, this evaluation is the first to quantitatively assess the intraoperative workload demand placed on the surgical teams during rTKA with a handheld RA surgical system compared to manual instrumentation. This multi‐centre analysis demonstrates that RA significantly reduces the perceived intra‐operative workload among surgical theatre staff, with pronounced decreases in NASA‐TLX scores for mental demand, physical demand and effort, all achieved without compromising perceived surgical performance.

In real‐world practice, the number of surgeries performed with manual instrumentation tends to reduce as surgeons' experience and confidence with the robotic technology increase, a trend consistent with this analysis. Surgeon choice is guided by individual preference and case requirements, reflecting natural variation in technique adoption. There remain exceptional cases where manual instrumentation is preferred or indicated, such as in extra‐articular deformities or the presence of intramedullary implants that could interfere with the placement of long‐stem components during arthroplasty.

The pattern of RA use varied, tending to be employed more frequently in both the least and more complex procedures rather than the most complex salvage cases. This is likely due to several factors: surgeons may opt for manual instrumentation in highly complex cases to avoid the added cognitive load of managing robotic technology; during early stages of the learning curve, surgeons preferentially use RA for simpler cases, gradually progressing to more complex cases as confidence grows.

Interpretation of the NASA‐TLX domain analysis revealed no significant differences between procedure types for performance, temporal demand, or frustration. This indicates the reduced workload associated with RA did not negatively affect perceived procedural performance, or operating pace. Subgroup analysis for the lead surgeons showed similar findings, with workload reductions achieved without compromising subjective performance outcomes.

Taken together, these findings among lead surgeons further support the consistent perceived reduction in subjective workload associated with RA. To further interpret these domain‐specific observations, OWSs were subsequently compared between groups.

OWS further contextualised these findings, aligning with domain‐specific results to indicate that RA not only reduces the sensed cognitive and physical demands but also lowers surgeons' overall perceived operative workload.

Overall, the consistent pattern of reduced subjective perceived workload across domains, and for lead surgeons specifically, suggests that the integration of RA meaningfully lessens the cognitive and physical demands of rTKA without compromising perceived performance. These findings provide important context for interpreting the broader implications of robotic technology on surgical ergonomics, efficiency and intraoperative focus.

The workload reduction findings align well with literature on RA in primary TKA and other surgical fields, where enhanced intraoperative feedback, surgical precision and ergonomics decrease surgeon fatigue and cognitive strain [4, 12, 19, 25, 28, 31]. While previous studies have largely focused on primary arthroplasty or general surgery, [4, 12, 19, 25, 28] this analysis provides novel evidence specific to the higher complexity of rTKA, a setting where cognitive and physical strain are amplified [22, 24]. The handheld RA surgical system converts a traditionally subjective, challenging procedure into a more objective, data‐driven workflow with real‐time 3D mapping and intraoperative feedback. This likely reduces intrinsic cognitive load and effort, as supported by significant reductions in the NASA‐TLX domains. This precision and predictability likely underpin the decreased mental and physical demands reported by surgeons. Key references addressing NASA‐TLX and workload demands includes studies on staff wellbeing [1, 2, 10], the mental effort demanded by more complex or novel procedures, [16] and workload analyses specific to orthopaedic surgery [4, 19] and knee arthroplasty, [12, 25, 28] including rTKA [22]. Our data highlights the favourable stress profile for surgeons when employing the handheld RA surgical system, consistent with reports that orthopaedic surgeons ‘feel at their best when they are operating’ [31]. The observed low frustration scores in both groups may reflect surgeon experience with rTKA and prior use of the handheld RA surgical system in primary knee surgeries.

### Clinical implications

These findings hold important clinical implications. Reduced cognitive load and physical strain could mitigate surgeon burnout and occupational injury risks, which are elevated in complex orthopaedic surgeries [1, 2, 5, 6, 9, 10, 18, 20, 21, 27, 30, 31, 32, 33]. Improved surgeon wellbeing may support consistent performance as revision procedures continue to increase globally [15, 23, 26, 29].

RA provides surgeons with objective intraoperative feedback previously unavailable, reducing cognitive load and uncertainty, supporting decision‐making, and enhancing surgical and staff performance. This feedback also may shorten learning curves and encourage the adoption of evolving technologies.

### Limitations

The foremost limitation of this analysis is the inherent subjectivity and retrospective nature of the NASA‐TLX instrument. As a post‐hoc, self‐reported measure of perceived workload, the NASA‐TLX is susceptible to recall bias and may be influenced by individual familiarity and prior experience with the handheld RA surgical system. This subjectivity constrains the interpretation of workload differences and limits direct objective validation of the findings. Most participating surgeons possessed considerable experience with robotic surgical systems, potentially diminshing the perceived workload reductions and limiting the generalisibility of results to less experienced surgeons or trainees.

Furthermore, the NASA‐TLX captures workload at a single static timepoint following each procedure, failing to account for potential intra‐individual fluctuations throughout surgery or longitudinal changes with learning and adaptation. This approach excludes real‐time workload assessment and may overlook nuanced variations during critical operative phases. The evaluation was also not designed to objectively measure or correlate workload perceptions with operative parameters such as procedure duration, motion tracking, or physiological stress markers, further limiting our ability to draw causal inferences about the relationship between subjective workload and objective performance metrics.

Additional limitations include the heterogeneity in sample distribution across surgical roles and centres, which restricted statistical analysis, particularly among smaller groups like trainee surgeons, scrub nurses and other theatre staff. Surgeon responses comprised only 41.5%–51.7% of surveys per technique, reflecting broad team input but limiting power for surgeon‐only subgroup analyses. Planned follow‐up analyses will prioritise role‐stratified modelling. Moreover, usage of the handheld RA surgical system was not protocol‐driven but based on individual surgeon preference, introducing potential confounding by indication (preference to use manual instrumentation for the more complex cases versus robotic‐assistance for less complex cases). Surgeons less experienced in rTKA tended to prefer manual instrumentation despite some familiarity with robotic surgical systems; however, variability in rTKA technique with manual instrumentation among surgeons also exists, potentially mitigating this confounding effect. There were 8.3% of cases performed in evenings when fatigue‐related workload elevations are likely highest. We did not conduct subgroup analyses by time of day, which may introduce residual confounding factors if evening cases disproportionately favoured one technique.

Lastly, the absence of institutional policy or formal guidelines governing the adoption of the handheld RA surgical system reflects real‐world variability but may limit the replicability of findings in settings with standardised protocols.

### Future directions

Preliminary findings support further investigation into the benefits of the handheld RA surgical system for rTKA. Future research should broaden to include objective ergonomic data (operative time, wearable motion or physiological sensors), longitudinal tracking of staff fatigue and recovery, and expanded role inclusion (trainee nurses, circulating staff, anaesthetists). Concurrent exploration of clinical outcomes such as radiological accuracy, and patient reported outcomes and satisfaction is recommended. The inclusion of NASA‐TLX in randomised controlled trials or prospective registries, alongside qualitative feedback from surgical teams, could strengthen evidence. Integration of workload metrics into routine quality assurance and staff planning tools is also recommended to optimise orthopaedic theatre function and team sustainability.

## CONCLUSION

In conclusion, this evaluation provides compelling real‐world evidence that the handheld RA surgical system significantly reduces perceived intraoperative workload during rTKA, as measured by the NASA‐TLX. Overall these findings support the integration of RA as a strategy to potentially enhance surgical comfort, reduce physical strain and enhance safety in revision knee arthroplasty.

## AUTHOR CONTRIBUTIONS

All authors contributed to the evaluation design and the acquisition, analysis and interpretation of the data. Catherine Whittall drafted the paper and revised it. All authors reviewed draft versions of the manuscript and provided recommendations or feedback. All authors have read and approved the final submitted version of this manuscript.

## CONFLICT OF INTEREST STATEMENT

All surgeons are paid consultants for Smith and Nephew.

## ETHICS STATEMENT

The authors have nothing to report.

## Data Availability

Research data are not shared.
